# Predicting fat cover in beef cattle to make on-farm management decisions: a review of assessing fat and of modeling fat deposition

**DOI:** 10.1093/tas/txae058

**Published:** 2024-04-11

**Authors:** Malcolm J McPhee

**Affiliations:** NSW Department of Primary Industries, Livestock Industries Centre, University of New England, Armidale, New South Wales, Australia

**Keywords:** adipose tissue, decision support tools, market specifications, rib fat, subcutaneous fat

## Abstract

Demands of domestic and foreign market specifications of carcass weight and fat cover, of beef cattle, have led to the development of cattle growth models that predict fat cover to assist on-farm managers make management decisions. The objectives of this paper are 4-fold: 1) conduct a brief review of the biological basis of adipose tissue accretion, 2) briefly review live and carcass assessments of beef cattle, and carcass grading systems used to develop quantitative compositional and quality indices, 3) review fat deposition models: Davis growth model (**DGM**), French National Institute for Agricultural Research growth model (**IGM**), Cornell Value Discovery System (**CVDS**), and BeefSpecs drafting tool (**BeefSpecsDT**), and 4) appraise the process of translating science and practical skills into research/decision support tools that assist the Beef industry improve profitability. The *r*^*2*^ for live and carcass animal assessments, using several techniques across a range of species and traits, ranged from 0.61 to 0.99 and from 0.52 to 0.99, respectively. Model evaluations of DGM and IGM were conducted using Salers heifers (n = 24) and Angus-Hereford steers (n = 15) from an existing publication and model evaluations of CVDS and BeefSpecsDT were conducted using Angus steers (n = 33) from a research trial where steers were grain finished for 101 d in a commercial feedlot. Evaluating the observed and predicted fat mass (**FM**) is the focus of this review. The FM mean bias for Salers heifers were 7.5 and 1.3 kg and the root mean square error of prediction (**RMSEP**) were 31.2 and 27.8 kg and for Angus-Hereford steers the mean bias were −4.0 and −10.5 kg and the RMSEP were 9.14 and 21.5 kg for DGM and IGM, respectively. The FM mean bias for Angus steers were −5.61 and −2.93 kg and the RMSEP were 12.3 and 13.4 kg for CVDS and BeefSpecsDT, respectively. The decomposition for bias, slope, and deviance were 21%, 12%, and 68% and 5%, 4%, and 91% for CVDS and BeefSpecsDT, respectively. The modeling efficiencies were 0.38 and 0.27 and the models were within a 20 kg level of tolerance 91% and 88% for CVDS and BeefSpecsDT, respectively. Fat deposition models reported in this review have the potential to assist the beef industry make on-farm management decisions on live cattle before slaughter and improve profitability. Modelers need to continually assess and improve their models but with a caveat of 1) striving to minimize inputs, and 2) choosing on-farm inputs that are readily available.

## Teaser

Fat deposition models have the potential to assist the beef industry make on-farm management decisions before slaughter.

## Introduction

Australia (**AU**) is the fourth largest beef exporter in the world behind Brazil, United States of America (**USA**), and India, with a gross beef export value of AU$9.2 billion in 2021 (Meat and Livestock Australia, 2022). Meeting market specifications, for beef cattle, in AU is an important industry issue for both domestic and foreign markets. The AU meat processing sector is highly regulated, by AUS-Meat (2018) and/or Meat Standards Australia (**MSA**) (Meat and Livestock Australia, 2013). Meat Standards Australia accredited abattoirs take into account all aspects of eating quality along the supply chain, where producers (i.e., ranchers in the USA context) are penalized if they do not meet market specifications related to fat distribution (i.e., subcutaneous fat thickness at Position 8 rump fat [**P8 rump fat**, mm] or 12th-rib fat thickness [12th-rib fat, mm] sites) and hot standard carcass weight (**HSCW**, kg). The United States Department of Agriculture (**USDA**) grading system (Hale et al., 2013) is similar to AU where they also have market specifications related to weight and fatness traits.

**Figure 1.**
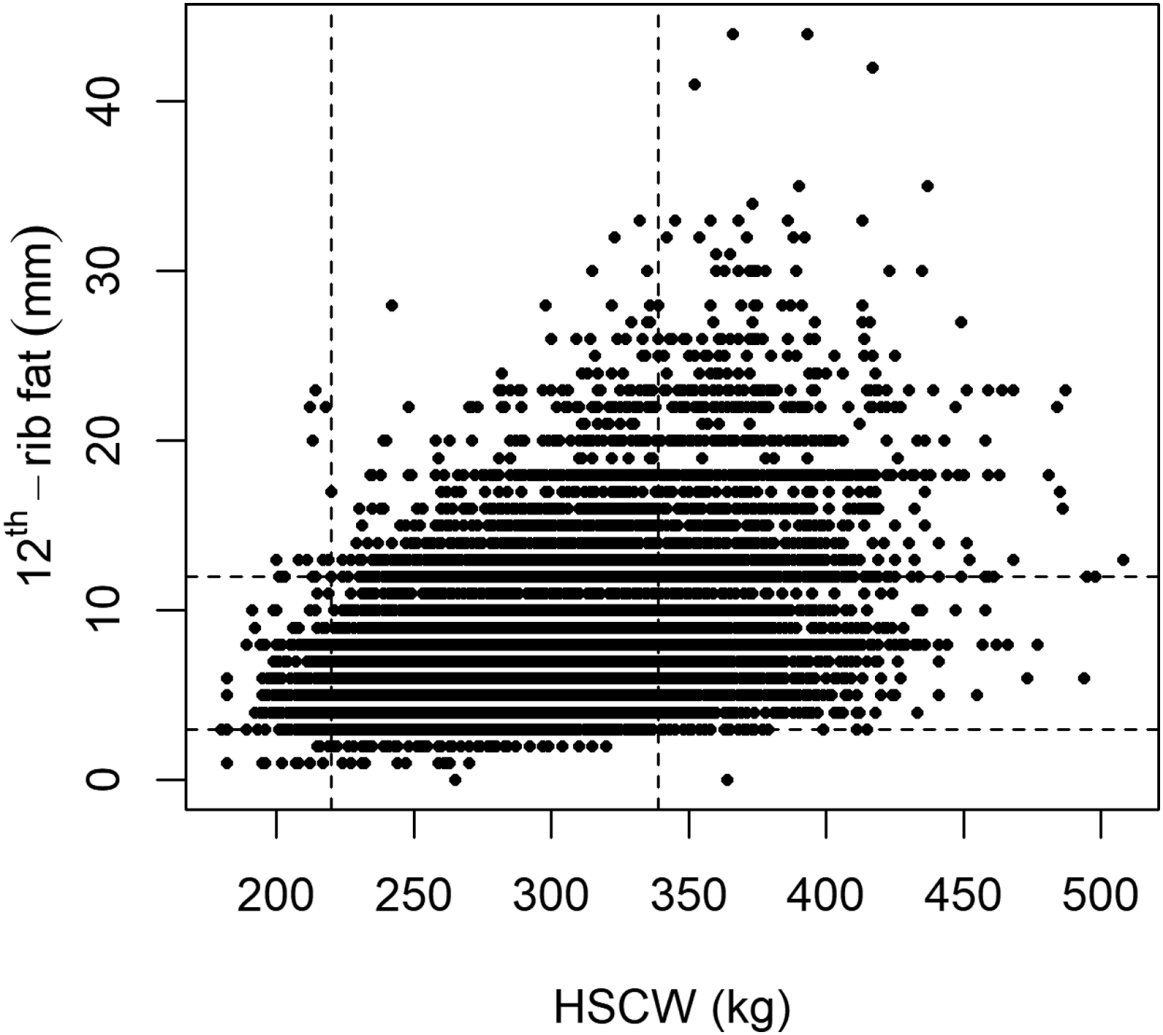
Market specifications of 12th-rib fat (mm) versus HSCW (kg) where dashed lines represent market specifications set at HSCW minimum = 220 kg and maximum = 339 kg and 12th-rib fat minimum = 3 mm and maximum = 12 mm. 17% and 13% noncompliance for HSCW and 12th-rib fat, respectively (McPhee and Walmsley, 2014).

Figure 1 illustrates an AU market specifications grid where the rectangular box represents the upper and lower market specifications of 12th-rib fat and HSCW. Market specifications can change when the beef industry is subject to price fluctuations owing to several factors including environmental conditions (e.g., droughts, floods, or fires), exotic disease outbreaks, pandemics, and/or changing market conditions. Therefore, the specified range for 12th-rib fat and/or HSCW, depicted in Figure 1, can either tighten or expand. Fat deposition models have the potential to play a critical role in assisting producers and managers make on-farm management decisions during periods when price fluctuations occur.

Fat deposition models require quality data and model developers need an understanding of the biology of fat deposition and the techniques used to make live and carcass assessments of beef cattle. Data from USDA and AU grading systems are also used in model development and evaluation. Therefore, the objectives of this paper were to 1) conduct a brief review of the biological basis of adipose tissue accretion covering the biology of fat, adipose tissue depots, number and size of adipocytes, and recruitment of adipocytes, 2) briefly review live and carcass assessments of beef cattle to report the accuracy of the techniques used for assessment and briefly review the USDA yield and MSA grades to state the factors/models of their grading systems, 3) review four fat deposition models: Davis Growth model (**DGM**), French National Institute for Agricultural Research growth model (**IGM**), Cornell Value Discovery System (**CVDS**), and BeefSpecs drafting tool (**BeefSpecsDT**), and lastly 4) discuss the differences between fat deposition models and the process of translating fat deposition models for either research or decision support tools (**DST**) that assist the beef industry improve profitability. The AU beef industry is the principal frame of reference. However, where applicable, beef production systems from other countries are referenced.

## Materials and Methods

This review consisted of compiling data from existing publications and databases therefore animal care and use committee approval was not obtained.

### Notation and Units

Special nomenclature used throughout this review are reported in Table 1.

**Table 1.** Description of notation and units

Item	Description	Units	Value
j	Increment for each fat depot: visceral intermuscular subcutaneous intramuscular	—	1 2 3 4
*f(X*_*1*_ *,…,X*_*p*_ *)*_*i*_	The *i*th model predicted (or simulated) value	—	—
*t*	Time	days	1 to DOF
*β*_*j*_(*t*)	Proportion of total body fat gain in each fat depot *j* at time *t* in days	—	—
ADS_MAXj_	Maximum adipocyte size for each fat depot adipose *j*	kg TG/g DNA	—
DNA_j_	Deoxyribonucleic acid	g DNA	—
DOF	Days on feed	Days	—
DST	Decision support tool	—	—
EBW	Empty body weight	kg	—
FAT	Total body fat	kg	—
FBW	Full body weight	kg	—
FM	Fat mass	kg	—
FFM	Fat-free mass	kg	—
F_j_	Fat in each fat depot *j*	kg TG	—
FP	Fraction of protein	—	—
FSIZ_j_	Adipocyte size for each fat depot adipose *j*	kg TG/g DNA	
HSCW	Hot standard carcass weight	kg	—
kFAT_j_	Fat parameter coefficient for each fat depot j	1/g DNA	—
KPH	Kidney, pelvic, and heart fat	%	—
LMY	Lean meat yield	%	—
ME	Metabolizable energy	MJ/d	—
M/D	Metabolizable energy density of feed	MJ ME/kg DM	—
MEI	Metabolizable energy intake	Mcal/kg DM	
NEg	Net energy for gain	Mcal/kg DM	—
NEm	Net energy for maintenance	Mcal/kg DM	—
P8 rump fat	fat measurement at the P8 rump site	mm	—
PROT	Protein	kg	—
RE	Retained energy	Mcal/d	—
TG	Triglycerides	—	—
*X*_*1*_ *,…,X*_*p*_	The *p*th input to the model	—	—
*Y*_*i*_	The *i*th observed or measured value	—	—
$\bar{Y}$	Mean of the observed (or measured) values	—	—

### Data for Model Evaluation

Model evaluations of the DGM and IGM were conducted using Salers heifers (n = 24) and Angus-Hereford steers (n = 15) reported in a study by Garcia et al. (2008) and model evaluations of CVDS and BeefSpecsDT were conducted using Angus steers (n = 33) from a research trial ran in a commercial feedlot (University of New England Tullimba feedlot, Kingston, AU). Predictions of fat mass (**FM**, kg) were compared to observed data where the observed FM values from the Garcia et al. (2008) study were either reported in a table or interpolated from figures (Table 4 and Fig. 5, Garcia et al., 2008).

**Figure 2.**
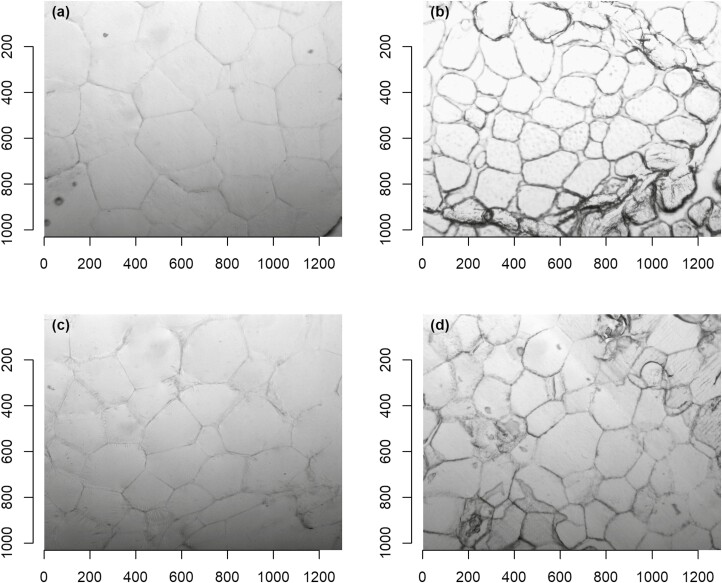
Image analysis of adipose tissue fat depots in Angus steers at 27 months of age (100× magnification): (a) mesenteric, kidney and channel, (b) subcutaneous, (c) intermuscular, and (d) intramuscular.

**Figure 3.**
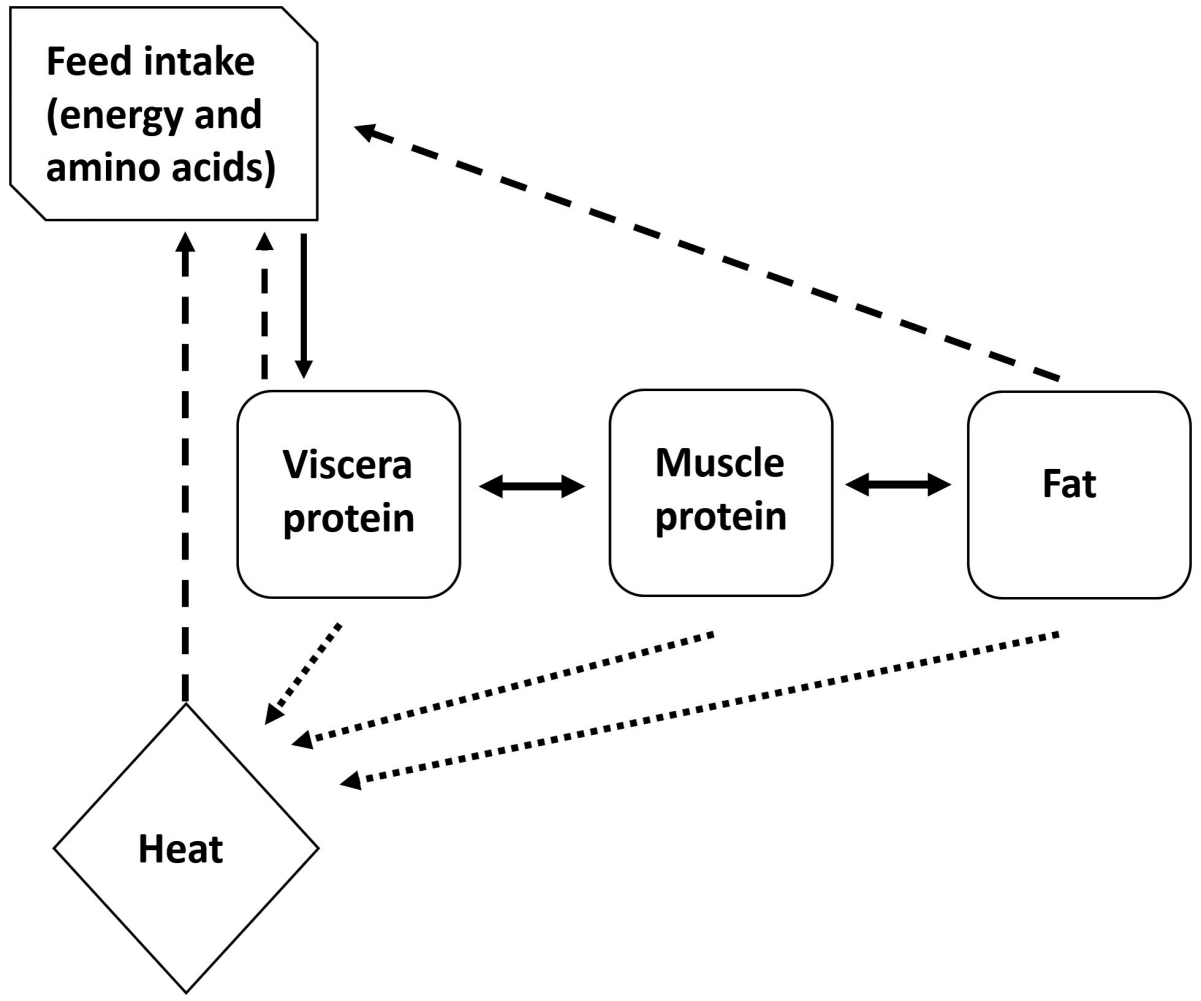
Schema describing the interactions between empty body protein and fat energy (Oddy et al., 2019).

**Figure 4.**
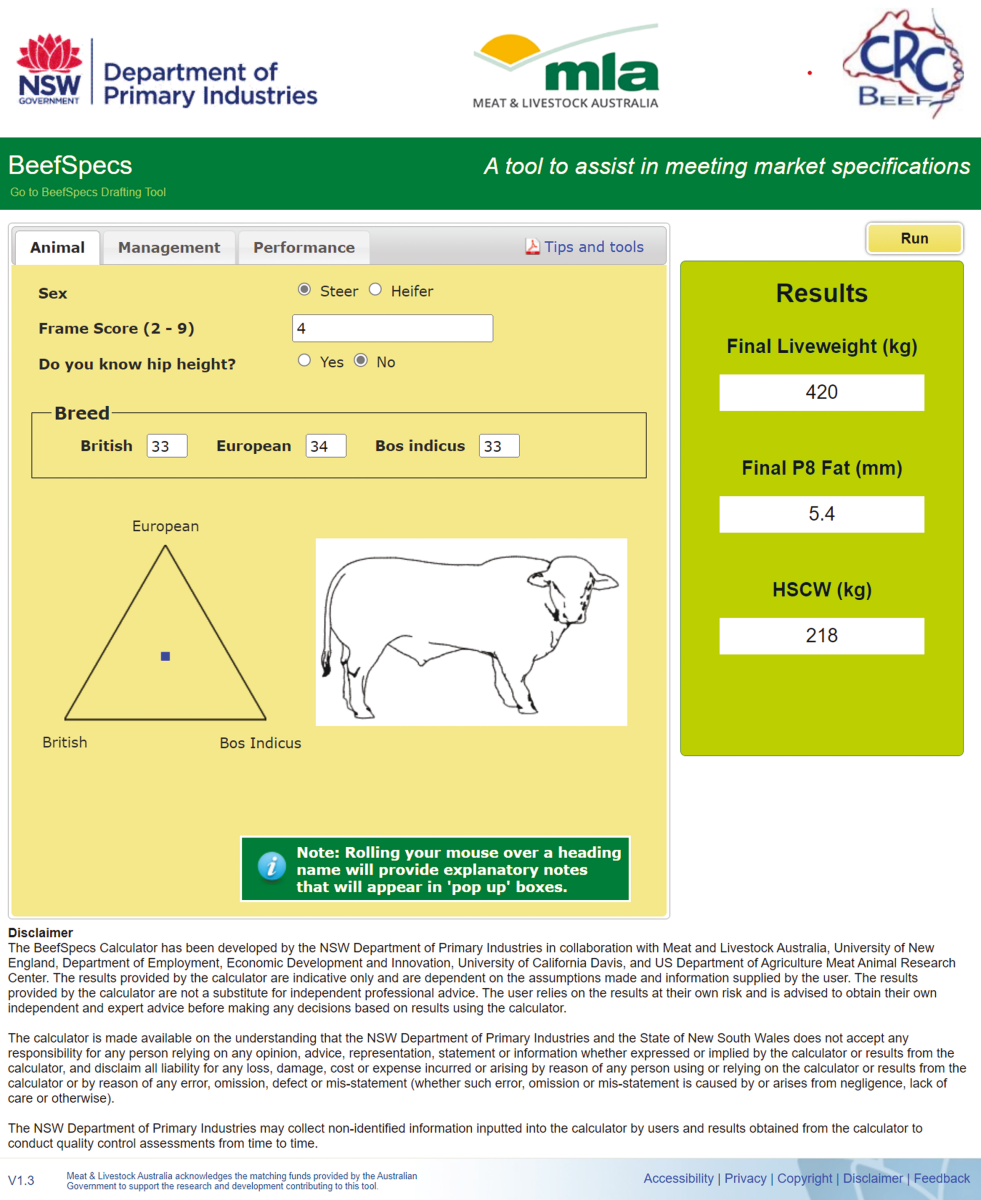
BeefSpecs fat calculator—Animal inputs tab.

On the other hand, the Angus steers observed FM were estimated from computed tomography (**CT**) scans of boned-out beef primals (e.g., rump). Primals were CT scanned using a Picker Ultra Z Spiral CT scanner (Philips Medical Imaging Australia, Sydney NSW). The X-ray tube operated at 130 kV and 100 mA. A pitch of 1.5, field of view of 500 mm, and cross-sectional thickness of 10 mm were used. All Angus carcasses from the research trial were MSA-graded.

A summary of the CVDS and BeefSpecsDT inputs is reported in Table 2. Additional inputs to CVDS include daily as-fed intake = 3.06 Mcal/kg DMI and adjusted final BW, and the BeefSpecsDT include ADG = 1.9 kg/d (a feedlot targeted commercial ADG), days on feed (**DOF**) = 101 d, finish = 1 for grain, sex = 1.3 for steers, breed = 100% British, and implant status = 0 for no implants.

**Table 2.** Summary statistics (n = 33) of data used for model evaluation of the BeefSpecs drafting tool and the Cornell Value Discovery System (CVDS)

Trait	Min.	Max.	Avg.	SD
Age^1^ (mo)	16.6	18.6	17.8	0.52
Initial BW (kg)	370	510	433	35.7
Initial P8 rump fat (mm)	2.00	8.00	5.06	1.52
Body condition score^1^	2.00	6.00	4.18	0.88
Frame score	4.00	8.00	5.85	1.06
Muscle score	6.00	9.00	7.30	0.95
Final BW^2^ (kg)	562	702	625	35.7
CT^3^ Fat mass (kg)	66.2	130	92.4	15.9

^1^Input to CVDS.

^2^Final BW based on a commercial growth rate of 1.9 kg/d over 101 d on feed. The final BW is used as the adjusted final BW input for CVDS.

^3^Computed tomography.

### Biological Basis of Adipose Tissue Accretion

Allen et al. (1976) describe the biology of fat in meat animals and Berg and Butterfield (1976) describe the patterns of accretion of muscle, bone, and fat using allometric principles (Huxley, 1932). Adipose tissue is one type of connective tissue, a collection of adipose cells (adipocytes) suspended in a matrix of connective tissue (Allen et al., 1976). The primary function of adipose tissue is energy storage so that metabolizable energy is available as required. Other functions of adipose tissue include insulator against heat loss, padding between organs, protection against bruising, vitamin storages (A, D, E, and K), structural membranes (e.g., phospholipids), and signaling (an interface between energy status and immune function). Initial programming is critical i.e., body fatness and fat distribution are genetically controlled (Allen et al., 1976).

Anatomically, four adipose tissue depots are recognized: 1) visceral fat (around kidneys, heart, pelvic (channel), and the intestinal tract (omental and mesenteric)), 2) intermuscular fat (between muscle), 3) subcutaneous fat (under the skin), and 4) intramuscular (within muscle) as described by Hammond (1955). Studies by Johnson et al. (1972) and Cianzio et al. (1982) have shown that the visceral adipose tissue depot is the first to fill with lipids. Hence, in the context of this review, the visceral adipose tissue depot is considered the first to fill.

While visceral, intermuscular, and subcutaneous adipocytes are typically 90 to 180 μm visceral adipocyte cells (i.e., perirenal fat) are generally larger (120 to 138 μm) than subcutaneous adipocytes (107 to 133 μm) (Allen, 1976). This is evident in Figure 2 summarizing the adipocyte size of adipose tissue depots (kg). Mesenteric, kidney, and channel, a subset of visceral adipocytes, appear to be larger than subcutaneous and intramuscular adipocytes, but similar in size to intermuscular adipocytes. Other studies (Cianzio et al., 1985; May et al., 1994; Lee et al., 2000) have found that that visceral adipocytes are larger than subcutaneous adipocytes in cattle over 15 months of age (Cianzio et al., 1985). Intramuscular adipocytes were the smallest, which typically range in size 40 to 110 μm (Cianzio et al., 1985; May et al., 1994; Lee et al., 2000).

The main factors that vary among genetically different animals is the number (i.e., hyperplasia) and size (i.e., hypertrophy) of intramuscular adipocytes. Adipocyte size is the primary variable influenced by feed intake and nutrition. It has been proposed that new intramuscular adipocytes may be recruited if needed (i.e., when enough cells exceeded the size threshold) after hyperplasia ceases. Harper et al. (2001) summarize the biochemical and genetic determinants of intramuscular deposition in cattle and Pethick et al. (2004) reviewed growth, development, and nutritional manipulation of marbling in cattle. More recently, Hausman et al. (2009) and Greenwood et al. (2019) reviewed the regulation of adipocyte development by nutrition and genetics, and the impacts of this on efficiency and meat quality.

### Live and Carcass Assessments of Beef Cattle

Quantitative data on live and carcass assessments utilizes a range of techniques. Either subjective or objective assessment techniques are used. Subjective assessments are a visual determination (Drennan et al., 2008), e.g., a manual assessment of P8 rump fat (NSW Department of Primary Industries, 2017). Subjective assessments can be affected by factors including the experience of the technician (i.e., livestock assessor), day-to-day variation within the technician’s assessments, contemporary group characteristics, and/or body fat. In contrast, objective assessments use technologies which are less prone to subjective errors.

Topel and Kauffman (1988) have described over 30 live and carcass compositional techniques in cattle, swine, and sheep and recently (Andrews, 2019) updated live and carcass cattle assessment techniques for the NSW Department of Primary Industries. The quantitative data on live cattle and carcasses are used to develop and evaluate mathematical fat deposition models.

#### Live assessments of beef cattle

Live beef cattle assessments of P8 rump fat, 12th-rib fat, eye muscle area (**EMA**, cm^2^), IMF (%), frame score, and muscle score are conducted at specific anatomical sites (Andrews, 2019). The most common technology used in AU for assessing fat (P8 rump fat [mm], 12th-rib fat [mm], and IMF [%]) and EMA is ultrasound scanning conducted by BreedPlan accredited technicians (Upton et al., 1999). More recently, live cattle objective assessments have been conducted using two- or three-dimensional (**2D**, **3D**) cameras. Two-dimensional assessments of biometric body measurements for Limousin cattle have been created from digital cameras (Ozkaya et al., 2016), 3D assessments of body condition score for dairy cows (Spoliansky et al., 2016), and 3D assessments of P8 rump fat and 12th-rib fat, and muscle score for Angus cattle (McPhee et al., 2017).

#### Carcass assessments of beef cattle

After slaughter by captive bolt stunning and exsanguination, the hide is removed. Some of the attached fat at the sites of measurement can be removed. Consequently, fat depth measurements on HSCW in commercial processing plants tend to be lower and more variable than those obtained by ultrasound scanning. In AU it is stated that beef primals (e.g., tenderloin, cube roll, striploin, and rump; described as closely trimmed retail cuts in the US) may be disqualified for an MSA grade (Meat and Livestock Australia, 2013) if fat distribution standards are inadequate i.e., 12th-rib fat < 3 mm, or P8 rump fat < 5 mm and/or have an area greater than 10 × 10 cm affected by hide puller damage.

Carcass fatness is assessed visually, or in some cases instrumentally, prior to entering the chiller. In AU, carcasses are assessed in the chiller by AUS-Meat trained assessors (AUS-Meat, 2018) and/or MSA graders (Meat and Livestock Australia, 2013) who make assessments of hot carcass P8 rump fat, meat and fat color, marbling, EMA, 12th-rib fat, and maturity of carcass. The methods and site of measurement/assessment are reported by Kempster et al. (1985) and Andrews (2019).

In addition to assessing marbling, a chemical analysis of IMF (%) is often taken when conducting research. Samples are taken from the LL site close to the 12/13th rib site. Measurements can be made either by solvent extraction using a Soxhlet apparatus or by near infrared spectrophotometry, calibrated against Soxhlet extraction, to estimate chemical intramuscular fat content (Perry et al., 2001).

Total carcass fat can be assessed either by 1) full dissections (Thompson et al., 1985; Perry and Arthur, 2000), 2) full chemical composition (Farid, 1991; Teixeira et al., 2017), 3) chemical composition using the 9th to 11th rib fat section (Hankins and Howe, 1946), 4) carcass specific gravity (Garrett and Hinman, 1969), 5) video image analysis (**VIA**) (Wassenberg et al., 1986; Rius-Vilarrasa et al., 2009), 6) CT scanning of carcasses (Romvàri et al., 1996; Alston et al., 2004, 2005; Vester-Christensen et al., 2009) and primal cuts (Prieto et al., 2010); magnetic resonance imaging (**MRI**), or 7) dual-energy X-ray absorptiometry (**DEXA**) (Scholz et al., 2013). Assessment of total body fat (i.e., whole body fat) using any of the above techniques to assess carcass fat also requires that omental and mesenteric fat from the rumen is added to total carcass fat along with kidney and pelvic fat (i.e., channel fat in an AU context).

Scholz et al. (2015) reviewed noninvasive methods for the determination of body and carcass composition in livestock and reported that CT is the most accurate method followed by MRI and then DEXA. Magnetic resonance imaging is used more so when studying smaller animals (e.g., mice or rabbits), therefore MRI is not discussed in this review. In relationship to CT scanning Romvàri et al. (1996) and Vester-Christensen et al. (2009) have reported the accuracy of lean meat (%) between fully dissected and CT data, on half sides of pigs.

Studies evaluating the DEXA technology have been conducted on the composition of beef rib sections (Mitchell et al., 1997); estimating lean meat yield (**LMY**, %) in sheep carcasses (Gardner et al., 2018); and cattle (Calnan et al., 2021). A prototype 3D imaging approach, a cheaper alternative to DEXA, to estimate LMY has also been trialed on beef carcasses (Alempijevic et al., 2021). In line with future AU research objectives, LMY is a universal measure irrespective of abattoir and market specification and can be a consistent feedback mechanism to producers. Alempijevic et al. (2021) reported LMY (%), accuracy/precision, between CT and 3D imaging technology on full sides of cattle using a 10-fold cross-validation technique. A recent review by Modzelewska-Kapituła and Jun (2022), on the application of computer vision systems, outlines additional applications of using image analysis technologies to assess meat products.

### Cattle Carcass Grading Systems Used to Develop Quantitative Compositional and Quality Indices

#### USDA yield grade

Hale et al. (2013) state that “the USDA, quality grade is a composite evaluation of factors that affect palatability of meat (tenderness, juiciness, and flavor)”. These factors include carcass maturity, firmness, texture, and color of lean, and the amount and distribution of marbling within the lean. Beef carcass quality grading is based on 1) degree of marbling and 2) degree of maturity. Graders evaluate the amount and distribution of marbling in the ribeye muscle at the cut surface after the carcass has been ribbed between the 12th and 13th ribs. Degree of marbling is the primary determination of quality grade. Hale et al. (2013) also stated that yield grades estimate the amount of boneless, primals from the high-value parts of the carcass (i.e., round, loin, rib, and chuck). The expected percentages of yield are outlined in Table 3.

**Table 3.** United States Department of Agriculture quality grade (Hale et al. 2013)

Yield grades	Expected yield (%)
1	>52.3
2	52.3 to 50.0
3	50.0 to 47.7
4	47.7 to 45.4
5	<45.5

#### Meat Standards Australia Grade

The MSA grade is based on empirical models that predict eating quality of individual beef muscles (Meat and Livestock Australia, 2013). Meat Standards Australia accredited abattoirs place MSA labels on beef primals to provide consumer assurance of eating quality at 3 levels: MSA 3, 4, and 5 in conjunction with a cooking method, for “roast, casserole, stir fry, thin slice, grill/pan fry, shabu-shabu, yakiniku, and corn” (Meat and Livestock Australia, 2013). The MSA grades of beef primals in the MSA grades booklet (Meat and Livestock Australia, 2013) state: 1) MSA grades are set from an analysis of consumer test results; 2) MSA grade standards are independent of all production factors; 3) MSA grade scores are composite, like USDA quality grades, of tenderness, juiciness, and flavor, but MSA grade scores also include overall liking scores, and 4) MSA grade scores reflect consumer judgment. Meat Standards Australia grades, like USDA quality grades, take into consideration the effect of marbling and ossification on eating quality in their grading. Meat Standards AU grades also consider cattle handling requirements, effect of tropical breeds, and pH on eating quality. Meat Standards AU grades currently do not report a yield percentage like the USDA yield grades. A summary of beef grading inputs for MSA and USDA quality grading systems is outlined in Table 4.

**Table 4.** Beef grading systems of MSA and USDA quality grade

Grading inputs	MSA	USDA
Tropical breed content	✓	
Hormonal growth promotants	✓	
Genotype	✓	
Carcass weight	✓	✓
Carcass conformation		✓
Ossification (maturity)	✓	✓
Meat texture		✓
Meat firmness		✓
Milk-fed veal	✓	
Hanging method	✓	
Marbling	✓	✓
Meat color	✓	✓
pH	✓	
Rib fat measurement	✓	✓
Ribeye area		✓
Fat color	✓	
Via saleyard	✓	
Cut aging	✓	
Cooking method	✓	
Individual cut	✓	

### Models of Fat Deposition

Previous sections have provided a brief overview of the biological basis of adipose tissue accretion and a review of live and carcass assessments and carcass assessments used to develop quantitative compositional and quality indices in cattle that are used to describe meat quality. It is apparent that post-death assessments do not empower producers to grow cattle to specifications, so the development of DSTs to manage growth to specified endpoints is required. The review now turns its attention to fat deposition models.

Modeling protein and fat deposition in beef cattle began with the studies of Simpfendorfer (1974) who developed relationships between EBW and body weight for *Bos tarus* steers. Fox and Black (1984) adjusted EBW models to accommodate genetically different sizes of cattle. As the power of desktop computers increased Williams and Jenkins (1998) developed a computer model to predict composition of EBW changes in cattle at all stages of maturity. Williams and Jenkins (2003) then developed a dynamic model of ME utilization for growing and mature cattle, with an objective to develop an aggregated model that could accurately predict daily gain when using daily ME intake.

As improvements and modifications were made to cattle models, researchers began to turn their attention to developing fat deposition models. The interest in developing fat deposition models emerged as the meat industry was seeking to reduce fat in both lamb (Thatcher and Gaunt, 1992) and pork (Brewer et al., 2001). For example, in the late 1990s both the AU and USA beef industries were faced with similar conflicting goals, of waste *versus* taste, when producing cattle to meet market specifications, with IMF (%) commanding a premium (Sainz and Hasting, 2000).

The following sections follow a chronological order of fat deposition models reported in studies by Oltjen et al. (1986), Hoch and Agabriel (2004a, 2004b), Tedeschi et al. (2004), and Walmsley et al. (2014) that are integrated within animal growth models either for research or for on-farm tactical decision making.

#### Davis growth model

The Oltjen et al. (1986) model, commonly referred to as the DGM, is an energetics model based on National Research Council equations (National Research Council, 1984). The DGM was originally developed for *Bos tarus* cattle but has also undergone improvements to include *Bos indicus* cattle (Sainz et al., 2006).

Studies by Oltjen et al. (1986); Di Marco et al. (1987); Bywater et al. (1988) and Oltjen et al. (2000) have detail on animal characteristics at a lower level of aggregation such as DNA accretion curves and protein to DNA ratios to set growth trajectories. Some have developed models that are more complex based on metabolic processes (Gill, 1984, 1996; France et al., 1987; Gill et al., 1989). “These complex models are based on the assumption that the distribution of nutrients in body tissues is controlled mainly by substrate availability, which follows the principles of saturation enzyme kinetics” (Baldwin, 1995).

The DGM uses a bottom-up conceptual approach i.e., cell represented by DNA then protein, fat, and finally animal. An extension of the DGM included the addition of four adipose tissue depots (kg): 1) intermuscular, 2) subcutaneous, 3) visceral, and 4) intramuscular, and was developed for researchers. The adipose tissue depots: subcutaneous, visceral, and intramuscular were partitioned by parameterizing Michaelis–Menten equations (McPhee et al., 2009). The intermuscular adipose tissue depot is the difference between total fat minus the sum of subcutaneous, visceral, and intramuscular adipose tissue depots. Thus, the intermuscular adipose tissue depot, the largest depot, accumulates errors from estimation of the other fat pools. Based on the meta-analysis data the proportion of total body fat partitioned into intermuscular, subcutaneous, visceral, and intramuscular adipose tissue depots were 57%, 20%, 17%, and 6%, respectively (McPhee et al., 2009).

The more general form of the DGM incorporates the concepts of hyperplasia and hypertrophy (i.e., DNA growth) based on mechanisms of growth to predict net protein and fat gain (synthesis—degradation) (equation 1):


$$\begin{array}{l} \frac{dX}{dt}=Synthesis-Degradation \end{array}$$ (1)

where *X* is a state variable for weight of protein or fat. In this study, the state variable for fat is an input to equation 3.

At the cell level (equation 2) adipose tissue depot parameters (β_*j*(*t*)_) were predicted as a proportion where 0 < β_*j*_ < 1 and Σβ_*j*_ = 1. Nomenclature of equations 1 to 4 are described in Table 1.


$$\begin{array}{l} \beta_{j\left(t\right)}=kFat_{j}\times DNA_{j\left(t\right)}\times \left[1-\left(\frac{FSIZ_{j}}{ADSMAX_{j}}\right)\right] \end{array}$$ (2)

First-order differential equations estimate fat deposition for each adipose tissue depot (*F*_*j*_)


$$\begin{array}{l} \frac{dF_{j}}{dt}=\beta_{j\left(t-1\right)}\times \frac{dFAT}{dt} \end{array}$$ (3)

for *j* = 1 to 4, intermuscular, subcutaneous, visceral, and intramuscular, respectively and $\frac{dFAT}{dt}$ (equation 1) is the rate of change of total fat in the carcass. In addition, at the animal level, each animal’s EBW is determined by


$$\begin{array}{l} \frac{dEBW}{dt}=\frac{1}{0.2201}\times \frac{dPROT}{dt}+\frac{dFAT}{dt} \end{array}$$ (4)

where $\frac{dPROT}{dt}$ and $\frac{dFAT}{dt}$ (equation 1) are the rates of change of protein and total fat in the carcass, respectively.

The DGM model: 1) integrates the net energy system to estimate gain of protein and fat and 2) accounts for variations attributable to initial body composition and mature size but does not always yield acceptable estimates of fat deposition, especially at high feed energy concentrations. Accurate estimates of fat deposition are not always possible because fat deposition is computed based on the amount of dietary energy remaining after energy requirements for maintenance and protein gain are satisfied. Therefore, errors in estimation of maintenance and protein gain are accumulated in fat. Generally, maintenance is variable and often greater in better-fed animals, or those coming off good diets, so the errors in underpredicting maintenance accumulate in fat predictions.

Studies by Oddy et al. (1997), Soboleva et al. (1999), and Oltjen et al. (2000) have continued the implementation and evaluation of physiological mechanisms (i.e., heat production in viscera protein, muscle protein, and fat) rather than substrate utilization models that relate to growth of body compartments. A review by Ferrell (1988), across a variety of experimental studies, indicated that the potential for variation in mass and energy expenditures of the liver and gastrointestinal tract had a major impact on total animal energy expenditure; conservatively, about 20% to 25% of the total could be attributed to each of those organ systems. Hence, modeling heat production at a tissue level in fat deposition models was considered a way forward to improving predictions of energy requirements and body composition in growing animals. Soboleva et al. (1999) in the development of a dynamic model of body composition in sheep included the modeling of heat production and more recently Oddy et al. (2019) integrated energy and protein transactions in the body (Figure 3). A sheep study by Dougherty et al. (2022) has also been conducted on building new tools for predicting performance and body composition of ruminants to include heat production where sheep may be growing fat and losing protein, or *vice versa*.

#### French National Institute for Agriculture Research growth model

The French National Institute for Agricultural Research (INRA, 1989) growth model was developed to evaluate energy requirements of growing beef cattle (Hoch and Agabriel, 2004a, b). The initial IGM growth model was based on empirical models developed by Robelin (1990) who fitted a Gompertz curve of body weight for different breeds. The underlying models have evolved and IGM became a dynamic and mechanistic model, based on biological processes to simulate beef cattle growth for different animal types under various nutritional conditions. The IGM is constructed on variations in proteins and lipids in carcass and non-carcass components. Therefore, there are 4 compartments in IGM: 1) protein in carcass, 2) protein in non-carcass, 3) fat in carcass, and 4) fat in non-carcass. Each of the four components is represented by a state variable and differential equations express the rate of change in each component as the difference between the rates of synthesis and degradation. Equations 5 and 6 are the protein synthesis and degradation in the carcass and non-carcass components


$$\begin{array}{l} SynProt_{i}=\alpha_{i\;\;\;}\times Prot_{i}\times \ln\left(\frac{Prot_{imax}}{Prot_{i}}\right)\times \frac{ME}{K_{ME}+ME} \end{array}$$ (5)

$$\begin{array}{l} DegProt_{i}=\gamma_{i}\times Prot_{i}\times \ln\left(\frac{Prot_{imax}}{Prot_{i}}\right) \end{array}$$ (6)

where α_*i*_ is the synthesis rate constant (d^−1^), Prot_*i*max_ is the maximum protein content (kg), Prot_*i*_ is the protein mass (kg) in the carcass or non-carcass tissues, and K_ME_ is the half-saturation coefficient (MJ/d) and γ_*i*_ is the degradation rate constant (d^−1^) and *i* is the daily time step. The lipid synthesis and degradation rates are identical to the protein synthesis and degradation equations 5 and 6, except that lipid max (Lip_*i*max_), which replaces Prot_*i*max_, is calculated as follows:


$$\begin{array}{l} Lip_{imax}=\left(Lip_{i0}+Lip_{i1}\times \left(\;\;\;\frac{Prot_{i}}{Prot_{imax}}\right)\;\;\;\right)\times W_{i} \end{array}$$ (7)

where Lip_*i*0_ and Lip_*i*1_ are coefficients of the linear relationship between maximum lipid content and physiological age and *W*_*i*_ is the weight of the body compartment (kg) as described by Hoch and Agabriel (2004a).

#### Cornell value discovery system

Tedeschi et al. (2004) developed the CVDS to improve individual cattle management of cattle fed in group pens. The CVDS is a progression of a growth model, a daily time (j) step model, developed by Fox and Black (1984). The total body fat initial estimate for the constraint *j* = 0 is as follows:


$$\begin{array}{lll} FAT_{j}=\; & (0.00054\times (SBW_{j=0}\times 0.891)\times 2\; & +0.037\times (SBW_{j=0}\times 0.891)-0.61) \end{array}$$ (10)

where **SBW**_***j***_ is shrunk body weight at time (*j*) = 0 and the accumulated total body fat for *j* > 0 is calculated as follows:


$$\begin{array}{l} FAT_{j}=(FAT_{j-1}+FIG_{j}\times EBW_{j}\times \;\;\;0.85) \end{array}$$ (11)

where FIG is fat in gain constrained for EBG_(*j* + *1*)_ > 0 and calculated as follows:


$$\begin{array}{l} FIG_{\left(\;j+1\right)}=0.123\times \frac{RE_{\left(\;j+1\right)}}{EBG_{\left(j+1\right)}}-0.154 \end{array}$$ (12)

where **RE** is retained energy for fat as described (equation 5, p. 185) by Tedeschi et al. (2004) and **EBG** is empty body gain (National Research Council, 2000).

#### BeefSpecs drafting tool

The BeefSpecs fat calculator (Figure 4) (http://beefspecs.agriculture.nsw.gov.au/) was initially developed from the DGM model but the underlying models in BeefSpecs were modified because dry matter intake (kg/d), a DGM input, was not generally assessed on grass finishing systems. The modifications made to the underlying BeefSpecs model (Walmsley et al., 2014) are based on the Keele et al. (1992) model that uses BW (kg) and ADG (kg/d) to predict fatness. Subsequently, the BeefSpecsDT (http://beefspecs.agriculture.nsw.gov.au/drafting) was developed that provides a visual assessment of compliance, with cattle either in or out of specifications, similar to those depicted in Figure 1. The BeefSpecs DST was developed for use by beef producers, on-farm managers, and livestock advisers.

**Figure 5.**
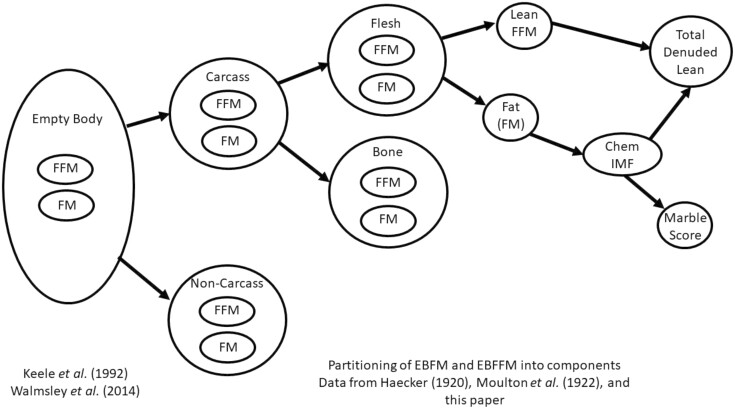
Partitioning of empty body fat-free mass (FFM) and FM into carcass and non-carcass, flesh, and bone to predict lean and fat components of a carcass (i.e., total denuded lean, chemical intramuscular (IMF) fat (%) and marble score) (McPhee et al., 2020).

Figure 5 shows the flow of partitioning the empty body fat-free mass (**FFM**) and FM into carcass, non-carcass, and flesh and bone components (McPhee et al., 2020). The FM is calculated as follows:


$$\begin{array}{l} FM=Flesh\times \frac{FleshFatPC}{100} \end{array}$$ (13)

The prediction of Flesh, FleshFatPC, and additional equations are described by McPhee et al. (2020). To produce industry-relevant outputs, two adipose tissue fat depots are converted to carcass characteristics: subcutaneous adipose tissue (kg) to 12th-rib fat (mm) and subsequently from 12th-rib fat (mm) to P8 rump fat (mm) (Walmsley et al., 2010b); and intramuscular adipose tissue (kg) to chemical intramuscular fat (%) (McPhee et al., 2020).

### Statistical Analysis

Comparison of observed *versus* predicted data were conducted using a customized procedure in R (R Core Team, 2024). Where feasible the following is reported: mean observed, predicted and bias, mean square error of prediction (MSEP, equation 14), root mean square error of prediction (**RMSEP**, equation 15), and MSEP decomposed into bias, slope, and deviance (Bibby and Toutenburg, 1977), modeling efficiency (equation 16) (Mayer and Butler, 1993), a dimensionless statistic, and a method proposed by Mitchell (1997) where the percentage of data points within upper and lower quality control limits are displayed on a plot of the residuals. The upper and lower quality control limits were set at ± 20 kg. Nomenclature of equations 14 to 16 are described in Table 1.


$$\begin{array}{l} MSEP=\frac{\overset{n}{\underset{i=1}{\sum }}{\left(Y_{i}-f{\left(X_{1},...,X_{p}\right)}_{i}\right)}^{2}}{n} \end{array}$$ (14)

$$\begin{array}{l} R\;MSEP=\surd MSEP \end{array}$$ (15)

$$\begin{array}{l} MEF=1-\frac{\sum_{i=1}^{n}{\left(Y_{i}-f{\left(X_{1},...,X_{p}\right)}_{i}\right)}^{2}}{\sum_{i=1}^{n}(Y_{i}-\bar{Y}{)}^{2}}. \end{array}$$ (16)

## RESULTS

### Live and Carcass Assessments of Beef Cattle

Examples of live animal and carcass assessments using ultrasound, 2D and 3D technologies, CT, DEXA, and VIA techniques for a range of traits are reported in Table 5. Table 5 includes the *r*^*2*^ and RMSEP from several studies where assessments from technologies *versus* observed data have been analyzed. The *r*^*2*^ for live animal assessments using ultrasound, 2D and 3D techniques, across a range of species and traits, ranged from 0.61 to 0.99 and the *r*^*2*^ for carcass assessment using CT, DEXA, VIA, and 3D techniques, across a range of species and traits ranged from 0.52 to 0.99 (Table 5).

**Table 5.** Examples of coefficient of determination (*r*^*2*^) and RMSEP, across species, for live and carcass assessment traits

Technique^1^	Species	Traits^2^	*n*	*r* ^*2*^	RMSEP	Reference
Live assessment						
Ultrasound	Cattle^3^	P8 rump fat^4^	13	0.87	—	Upton et al. (1999)
		Rib fat^D^	13	0.84	—	“
		EMA^D^	13	0.65	—	“
		IMF^D^	13	0.71	—	“
Ultrasound	Cattle^5^	P8 rump fat^4^	16	0.87	—	Upton et al. (1999)
		Rib fat^4^	16	0.88	—	“
		EMA^4^	16	0.85	—	“
		IMF^D^	16	0.77	—	“
Ultrasound	Sheep	12 and 13th ribs	96	0.67	2.77 mm	Thériault et al. (2009)
2D	Cattle	HH	56	0.97	1.80 cm	Ozkaya et al. (2016)
3D	Cows	HH	156	0.75	3.44 cm	McPhee et al. (2017)
3D	Steers	HH	79	0.90	2.89 cm	“
3D	Cows	MS	156	0.74^6^	80%^7^	“
3D	Steers	MS	79	0.79^6^	83%^7^	“
3D	Cows	P8 rump fat^8^	156	0.94	1.54 mm	“
3D	Steers	P8 rump fat^8^	79	0.71	1.00 mm	“
3D	Cows	BCS	20^9^	0.75	0.26^10^	Spoliansky et al. (2016)
3D	Cows^11^	BCS-lateral	55	0.63	0.16	Martins et al. (2020)
3D	Cows^11^	BCS-dorsal	55	0.61	0.17	“
Carcass assessment						
CT	Pigs	Lean meat	60	0.99	0.56%	Romvàri et al. (1996)
	Pigs	Lean meat	299	0.99	83.6 g	Vester-Christensen et al. (2009)
DEXA	Beef	9, 10, 11th rib section fat	80	0.94	—	Mitchell et al. (1997)
	Pigs	Shoulder	262	0.76	—	Mitchell et al. (2003)
	Sheep	Lean meat	607	0.69	1.69%	Gardner et al. (2018)
	Pigs	Leg fat mass	9	—	5.06 kg	Kipper et al. (2019)
	Cattle	Lean meat	51	0.89	2.31%	Calnan et al. (2021)
VIA	Sheep	Lean meat	360	0.52	—	Hopkins et al. (2004)
3D	Cattle	Lean meat	119	0.69	3.91%	Alempijevic et al. (2021)

^1^3D = 3 dimensional; CT = computed tomography; DEXA = dual-energy X-ray absorptiometry; VIA = video image analysis; MRI = magnetic resonance imaging.

^2^HH = hip height; MS = muscle score; BCS = body condition score.

^3^Observed May 1997: mean (n = 3) P8 rump fat carcass assessment.

^4^Observed: carcass assessment.

^5^Observed June 1998: mean (n = 3) P8 rump fat carcass assessment.

^6^Cohen’s kappa (κ) statistic (Cohen, 1960).

^7^Correctly classified instances.

^8^Observed: ultrasound scanned.

^9^11,824 images.

^10^Average mean absolute error.

^11^Holstein heifers (n = 27) and lactating cows (n = 28).

### Model Evaluation

#### Davis growth model and French National Institute for agriculture research growth model

The mean bias in Table 6 indicates that both the DGM and IGM under-predicted the mean of the FM observations by ≤7.5 kg for the Salers heifers and over-predicted the mean of the FM observations by ≤10.5 kg for the Angus-Hereford steers. The RMSEP was higher than 20 kg for Salers heifers with the DGM and IGM models and Angus-Hereford steers for IGM but were lower than 10 kg for the Angus-Hereford steers with DGM (Table 6).

**Table 6.** Model evaluation of observed *versus* predicted fat mass (kg) for Salers heifers, and Angus-Hereford (AH) steers, with Davis Growth Model (DGM), and French National Institute for Agricultural Research Growth Model (IGM), and Angus steers with Cornell Value Discovery System (CVDS), and BeefSpecs drafting tool (BeefSpecsDT)

	Salers heifers		Angus-Hereford steers		Angus steers	
	DGM^1^	IGM^1^	DGM^2^	IGM^2^	CVDS^3^	BeefSpecsDT
*n*	24	24	15	15	33	33
Mean observed, kg	160	160	116.7	116.7	92.4	92.4
Mean predicted, kg	152.5	158.7	120.0	127.2	98.0	95.3
Mean bias, kg	7.5	1.3	−4.0	−10.5	−5.61	−2.93
MSEP^4^	970.4	772.9	83.6	462.9	151	179
Root-MSEP, kg	31.2	27.8	9.14	21.5	12.3	13.4
Bias, %	—	—	—	—	20.8	4.79
Slope, %	—	—	—	—	11.7	4.21
Deviance, %	—	—	—	—	67.5	91.0
Modeling Eff	—	—	—	—	0.38	0.27
WCL^5^, %	—	—	—	—	91	88

^1^Mean observed, and bias interpolated from Figures 4 and 5, and MSEP reported in Table 4 for dataset CG-P4 (Garcia et al. 2008). Mean predicted is calculated (mean observed—mean bias).

^2^Mean observed reported in Table 5 by Garcia et al. (2008) for dataset CA-CA (Sainz et al., 1995). Mean bias interpolated from Figure 5, and MSEP reported in Table 4 for dataset CG-P4 by (Garcia et al., 2008). Mean predicted is calculated (mean observed—mean bias).

^3^Adjusted empty body fat to FleshFatPC (Equation (8), McPhee et al. (2020)) to calculate fat mass.

^4^MSEP = mean square error of prediction error, Bias = MSEP decomposed into error due to overall bias of prediction; Slope = MSEP decomposed into error due to deviation of the regression slope from unity, Deviance = MSEP decomposed into error due to the deviance variation.

^5^WCL = within upper and lower control limits.

#### Cornell Value Discovery System and BeefSpecs drafting tool

**Figure 6.**
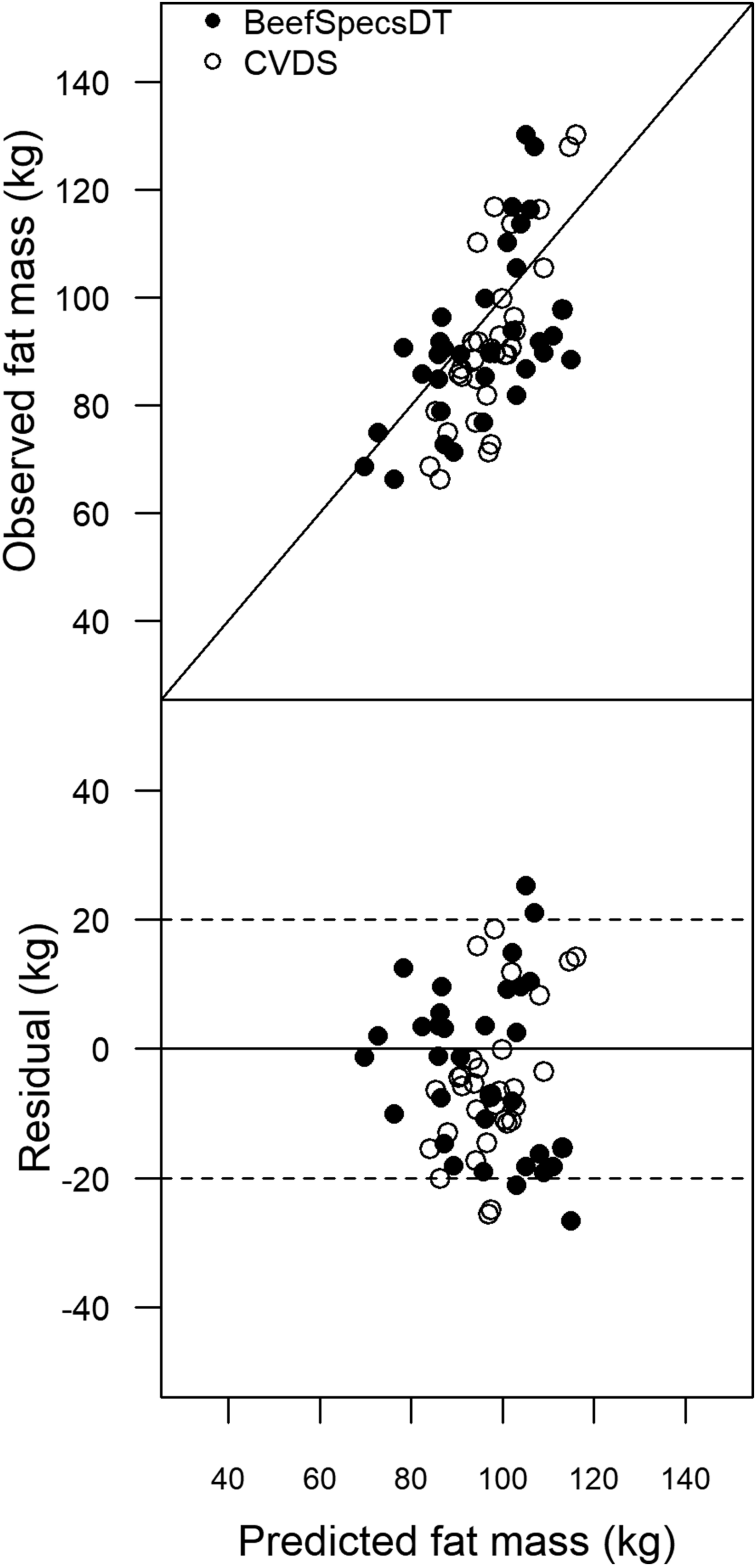
Scatter plots of BeefSpecs drafting tool (BeefSpecsDT) and Cornell Value Discovery System (CVDS) of the observed (CT) versus predicted fat mass (kg) where the solid black line is the 1:1 relationship and the plot of the residuals (observed—predicted) versus the predicted fat mass (kg) where the dashed lines are the upper and lower control limits of 20 kg, and the solid line represents the residuals = 0.

The mean bias in Table 6 indicates that both the CVDS and BeefSpecsDT over-predicted the mean of the FM observations by <6 kg. The RMSEP of FM was lower than 15 kg for both CVDS and BeefSpecsDT. The decomposition of the MSEP shows that CVDS had bias and slope and for the BeefSpecsDT it was <5%; most of the decomposition error, contained in the predictions, were due to deviance for both the BeefSpecsDT and CVDS. The MEFs for both models were positive indicating that the models were following a 1:1 (y = x) relationship, also shown in Figure 6. The percentage of residuals (Figure 6), within the upper and lower quality control limits of 20 kg, were 91 and 88% for CVDS and BeefSpecsDT, respectively.

## Discussion

### Similarities and Differences

Similarities and differences between the DGM, IGM, CVDS, and BeefSpecsDT are reported in Table 7. All fat deposition models reviewed in this study include empirical equations and structural differences between protein and lipid. It is not surprising that all models are driven, to some degree, by empirical equations and structural differences between protein and lipid because linear and nonlinear responses are reported extensively. The easiest step in a modeling exercise is to implement linear or nonlinear relationships reported in peer reviewed publications into models e.g., predicting initial conditions using empirical equations is very common.

**Table 7.** Similarities and differences between the DGM, the French National Institute for Agricultural Research growth model (IGM), the Cornell Value Discovery System (CVDS), and BeefSpecs drafting tool (BeefSpecsDT)

Description	DGM	IGM	CVDS	BeefSpecsDT
Average daily gain drives the model				✓
Dry matter intake drives the model	✓	✓	✓	
Carcass and non-carcass components		✓		✓
Dynamic model (i.e., behavior over time)	✓	✓	✓	✓
Empirical relationships	✓	✓	✓	✓
Meat quality traits	✓	✓	✓	✓
On-farm decision support tool			✓	✓
Research model	✓	✓		
Structural differences between body protein and lipid	✓	✓	✓	✓
Synthesis and degradation of protein	✓	✓		

### Davis Growth Model and French National Institute for Agriculture Research Growth Model

The interpolated FM from the study by Garcia et al. (2008), reported in this review, between the DGM and IGM, illustrate that both models had some difficulty in predicting the FM for Salers heifers and the IGM model for Angus-Hereford steers. However, the DGM predicted FM with a good level of accuracy (RMSEP < 10 kg) for Angus-Hereford steers (Table 6). Similarities exist between IGM and DGM e.g., both have developed models with structural differences between body protein and lipid contents and differential equations of synthesis and degradation for protein and fat. However, IGM makes a distinction between proteins in both the body and viscera. The main difference between IGM and DGM is that instead of combining lipid components into a single compartment by summing all adipose tissues, Hoch and Agabriel (2004a, 2004b) separated carcass and non-carcass lipids to simulate the growth and composition of the whole body.

The comparative analysis by Garcia et al. (2008) found that both model structures and equations of IGM and DGM produce adequate predictions of protein accretion. The study by Garcia et al. (2008) also found that 1) future improvements in prediction of heat production during periods of feed restriction were required for the DGM and 2) mathematical formulation of feed energy and utilization for fat synthesis was required to improve model sensitivity of ME intake in IGM (Garcia et al., 2008).

### Cornell Value Discovery System and BeefSpecs drafting tool

The model evaluation, in this study, between CVDS and BeefSpecsDT found that both models adequately predict FM (Table 6). However, the calculation of FM for CVDS (Equation 11) and BeefSpecsDT (Equation 13) are very different. The analysis between CVDS and BeefSpecsDT was achieved by translating the CVDS EBW fat (%) to the BeefSpecs FleshFatPC (Equation (8), McPhee et al. (2020)). The inputs between CVDS and BeefSpecsDT are also different, the CVDS inputs include as-fed intake (3.06 Mcal/kg DMI) and adjusted final BW (initial BW + DOF × ADG; DOF = 101 and ADG = 1.9 kg.d) and the BeefSpecsDT inputs include initial P8 rump fat (mm). BeefSpecsDT final BW is equivalent to CVDS’s adjusted final BW. The CVDS is strongly focused on grain finishing systems and the BeefSpecsDT is strongly focused on grass finishing systems. Even though, the BeefSpecsDT is focused on grass finishing systems it can, as indicated in Table 6, adequately predict FM. The decision to use intake as the driver for CVDS and ADG for the BeefSpecsDT has largely been driven by the finishing systems (grain or grass) within the USA (grain finishing) and AU (grass finishing) markets.

### Translating Fat Deposition Models into Decision Support Tools: From Theory to Practice

Modeling beef cattle fat deposition to assist producers to make on-farm management decisions requires a unique set of skills in 1) the biological basis of adipose tissue accretion; 2) assessing live beef cattle and carcass beef traits; and 3) skills in mathematical modeling to translate the factors affecting fat deposition from theory to practice. All models reported in this review have translated theoretical models into either research models or practical on-farm DST.

Those with the skills to develop research models and on-farm DST need to develop good communication skills with those who use DST. Developing communication between producers and researchers is imperative. The BeefSpecs team engaged producers and extension staff in the development of the BeefSpecsDT (Walmsley et al., 2014).

Baldwin, over many years, as reported by Sainz (2013), used a systems approach to develop a mechanistic model of dairy cow metabolism, (Baldwin et al., 1987). On-farm participatory research (McCown, 2001) is another example of a systems approach where researchers with the theoretical knowledge and modeling skills collaborate with practitioners. Translating the science behind fat deposition, skills in assessing live cattle, carcass traits, and mathematical modeling skills into an on-farm DST is effectively an on-farm participatory process. Both McCown (2001) and Sainz (2013) highlight that a systems approach points towards an environment where there are two complimentary strategies where the systems framework for research includes the reality of the farm as a human system through: 1) ‘systems thinking’ - new knowledge of social concepts, and 2) “systems practice”—involving producers and/or managers in the research. The strategies “systems thinking” and “systems practice” are integral components of the translation process from conceptual thinking, collecting data, and building mathematical models through to the development of a DST. The process of translating the science behind fat deposition, assessing beef cattle, and modeling fat deposition involves producers, advisors, scientists, and mathematicians.

The BeefSpecs calculator (Walmsley et al., 2014), BeefSpecsDT (Walmsley et al., 2010a) and the BeefSpecs feedlot optimization tool (Mayer et al., 2013) have all been developed specifically for the AU cattle industries. The BeefSpecsDT was developed to assist producers meet market specifications and reduce the losses incurred when they fail to meet market specifications. Figure 1 illustrates an overall 30% loss in not meeting market specification of 12th-rib fat and HSCW. But even more is lost if feeding costs are taken into consideration. Brazilians have also developed a system specific to their market called BeefTrader (Albertini et al., 2017) that optimizes the economical endpoint for feedlots and meat packers. More recently, the BeefSpecsDT has been integrated with a real-time 3D technology, that takes approximately 30 to 40 s to assess hip height and P8 rump fat when inducting cattle into a pasture or grain finishing system, called CattleAssess3D (McPhee et al., 2023). The real-time assessment of hip height and P8 rump fat from 3D images is seen as a leap forward in saving on-farm labor and associated costs when assessing cattle to make on-farm management decisions. However, the skills of assessing live cattle and carcasses should never be lost amongst the wave of precision agriculture and the plethora of automated measurement and prediction technologies.

## Conclusion

This paper has 1) briefly reviewed the fundamental biological basis of adipose tissue accretion, 2) briefly reviewed live and carcass assessment techniques of beef cattle, and cattle carcass grading used to develop quantitative compositional and quality indices, and 3) reviewed models of fat deposition. Biology of fat accretion and practical skills are all required to build DST that assist producers meet market specifications. There is no right or wrong model; errors occur in all models. However, each model has the potential to improve our understanding of the science and biology behind fat deposition models. The practical aspects of assessing live and carcass traits are not going to go away. Fat deposition modelers are beholden to those who have practical skills in assessing cattle and researchers who produce quality data on fat deposition in beef cattle. We need to be active in continually training younger staff to develop the skills needed to assess beef cattle.

Developers of fat deposition models and DST need to bear in mind the trade-offs between greater accuracy and precision on the one hand and the practicality and usefulness of the DST on the other. For example, the use of -omics (genomics, transcriptomics, proteomics, and metabolomics) technologies e.g., the expression of mRNA (Dumas et al., 2008) might greatly improve model predictions, but if on-farm inputs are not available to drive such a model its usefulness would be limited. However, large amounts of -omics data, at the appropriate level of aggregation, are required to clearly define genetic differences in phenotype and support the future development of dynamic models. These data are often difficult and expensive to obtain. The process of model development and the unending improvement to models follows the Kaizen principles (Imai, 1986) of ‘continuous improvement’ but with a caveat of 1) striving to minimize inputs, and 2) choosing on-farm inputs that are readily available.
